# Supplementary material for: One-instrument, objective microsatellite instability analysis using high-resolution melt
Source: PLoS One. 2024 Apr 25;19(4):e0302274. doi: 10.1371/journal.pone.0302274 (PMC11045061; doi:10.1371/journal.pone.0302274)
Supplement: S1 Table — (DOCX) [file pone.0302274.s001.docx]

**S1 Table. PlentiPlex™ MSI PentaBase Panel primers.**

| **Locus** | **Fluorophore** | **Forward primer** | **Reverse primer** |
| --- | --- | --- | --- |
| **BAT25** | PentaGreen | 5’-TCGCCTCCAAGAATGTAAGT-3’ | 5’-TCTGCATTTTAACTATGGCTC-3’ |
| **BAT26** | PentAltRed | 5’-TGACTACTTTTGACTTCAGCC-3’ | 5’-AACCATTCAACATTTTTAACCC-3’ |
| **NR22** | PentaGreen | 5’-GAGGCTTGTCAAGGACATAA-3’ | 5’-AATTCGGATGCCATCCAGTT-3’ |
| **NR24** | PentAltYellow | 5’-CCATTGCTGAATTTTACCTC-3’ | 5’-ATTGTGCCATTGCATTCCAA-3’ |
| **MONO27** | PentAltRed | 5’-AGTGAGCTGTGATTGCACT-3’ | 5’-CTACTGAAATGTAAGCTAGGAC-3’ |
