# Supplementary material for: One-instrument, objective microsatellite instability analysis using high-resolution melt
Source: PLoS One. 2024 Apr 25;19(4):e0302274. doi: 10.1371/journal.pone.0302274 (PMC11045061; doi:10.1371/journal.pone.0302274)
Supplement: S3 Table — (DOCX) [file pone.0302274.s003.docx]

**S3 Table. Software settings for MSI analysis on BaseTyper™ using paired samples and (universal reference).** RFU: relative fluorescence unit.

|  | **Normalization Temperature (°C)** | | | |  |  |  |
| --- | --- | --- | --- | --- | --- | --- | --- |
|  | **Initial** | | **Final** | |  |  |  |
|  | **From** | **To** | **From** | **To** | **Method** | **Temp Shift (RFU)** | **Threshold (RFU)** |
| **BAT25** | 38 | 39 | 63 | 64 | Ratio | 0.1 | -0.04 |
| **BAT26** | 43 | 44 | 65 | 66 | Ratio | 0.1 | -0.03 |
| **NR22** | 44 | 45 | 66 | 67 | Ratio | 0.1 | -0.03 |
| **NR24** | 44 | 45 | 66 | 67 | Ratio | 0.1 | -0.04 |
| **MONO27** | 45 | 46 | 67 | 68 | Ratio | 0.1 | -0.04 |
