# Supplementary material for: One-instrument, objective microsatellite instability analysis using high-resolution melt
Source: PLoS One. 2024 Apr 25;19(4):e0302274. doi: 10.1371/journal.pone.0302274 (PMC11045061; doi:10.1371/journal.pone.0302274)
Supplement: S4 Table — (DOCX) [file pone.0302274.s004.docx]

**S4 Table. Results from development cohort using paired samples and universal reference.**

|  | **Sensitivity %**  **[95% CI]** | **Specificity %  [95% CI]** | **True positive** | **False negative** | **False positive** | **True negative** | **Total** |
| --- | --- | --- | --- | --- | --- | --- | --- |
| **Paired** |  |  |  |  |  |  |  |
| BAT25 | 96.97  [84.68; 99.46] | 100.00  [97.54; 100.00] | 32 | 1 | 0 | 152 | 185 |
| BAT26 | 100.00  [89.85; 100.00] | 100.00  [97.52; 100.00] | 34 | 0 | 0 | 151 | 185 |
| NR22 | 100.00  [89.28; 100.00] | 100.00  [97.55; 100.00] | 32 | 0 | 0 | 153 | 185 |
| NR24 | 100.00  [89.57; 100.00] | 100.00  [97.54; 100.00] | 33 | 0 | 0 | 152 | 185 |
| MONO27 | 100.00  [89.57; 100.00] | 100.00  [97.54; 100.00] | 33 | 0 | 0 | 152 | 185 |
| **Universal** |  |  |  |  |  |  |  |
| BAT25 | 100.00  [89.57; 100.00] | 98.03  [94.36; 99.33] | 33 | 0 | 3 | 149 | 185 |
| BAT26 | 94.12  [80.91; 98.37] | 100.00  [97.52; 100.00] | 32 | 2 | 0 | 151 | 185 |
| NR22 | 100.00  [89.28; 100.00] | 92.16  [86.79; 95.46] | 32 | 0 | 12 | 141 | 185 |
| NR24 | 100.00  [89.57; 100.00] | 100.00  [97.54; 100.00] | 33 | 0 | 0 | 152 | 185 |
| MONO27 | 100.00  [89.57; 100.00] | 97.37  [93.43; 98.97] | 33 | 0 | 4 | 148 | 185 |
