# Supplementary material for: One-instrument, objective microsatellite instability analysis using high-resolution melt
Source: PLoS One. 2024 Apr 25;19(4):e0302274. doi: 10.1371/journal.pone.0302274 (PMC11045061; doi:10.1371/journal.pone.0302274)
Supplement: S5 Table — (DOCX) [file pone.0302274.s005.docx]

**S5 Table. Sensitivity and specificity using paired samples and universal reference for three extraction methods and two real-time PCR instruments.**

|  |  | **BAT25** | **BAT26** | **NR22** | **NR24** | **MONO27** |
| --- | --- | --- | --- | --- | --- | --- |
| **MyGo Pro  QIAamp** | **Paired** |  |  |  |  |  |
|  | Specificity % | 100.00  [77.19;100.00] | 100.00  [77.19;100.00] | 100.00  [77.19;100.00] | 100.00  [77.19;100.00] | 100.00  [77.19;100.00] |
|  | Sensitivity % | 100.00  [87.54;100.00] | 100.00  [87.54;100.00] | 100.00  [87.54;100.00] | 100.00  [87.54;100.00] | 100.00  [87.54;100.00] |
|  | **Universal** |  |  |  |  |  |
|  | Specificity % | 92.3  [66.69;98.63] | 100.00  [77.19;100.00] | 100.00  [77.19;100.00] | 100.00  [77.19;100.00] | 100.00  [77.19;100.00] |
|  | Sensitivity % | 100.00  [87.54;100.00] | 100.00  [87.54;100.00] | 100.00  [87.54;100.00] | 100.00  [87.54;100.00] | 100.00  [87.54;100.00] |
| **MyGo Pro**  **BaseRelease™** | **Paired** |  |  |  |  |  |
|  | Specificity % | 100.00  [77.19;100.00] | 100.00  [77.19;100.00] | 100.00  [77.19;100.00] | 100.00  [77.19;100.00] | 100.00  [77.19;100.00] |
|  | Sensitivity % | 96.3  [81.71;99.34] | 100.00  [87.54;100.00] | 100.00  [87.54;100.00] | 100.00  [87.54;100.00] | 100.00  [87.54;100.00] |
|  | **Universal** |  |  |  |  |  |
|  | Specificity % | 53.9  [29.14;76.79] | 92.3  [66.69;98.63] | 100.00  [77.19;100.00] | 84.6  [57.77;95.67] | 100.00  [77.19;100.00] |
|  | Sensitivity % | 100.00  [87.54;100.00] | 100.00  [87.54;100.00] | 100.00  [87.54;100.00] | 100.00  [87.54;100.00] | 100.00  [87.54;100.00] |
| **MyGo Pro**  **BasePurifier™** | **Paired** |  |  |  |  |  |
|  | Specificity % | 100.00  [77.19;100.00] | 100.00  [77.19;100.00] | 100.00  [77.19;100.00] | 100.00  [77.19;100.00] | 100.00  [77.19;100.00] |
|  | Sensitivity % | 100.00  [87.54;100.00] | 100.00  [87.54;100.00] | 100.00  [87.54;100.00] | 100.00  [87.54;100.00] | 100.00  [87.54;100.00] |
|  | **Universal** |  |  |  |  |  |
|  | Specificity % | 76.9  [49.74;91.82] | 92.3  [66.69;98.63] | 100.00  [77.19;100.00] | 84.6  [57.77;95.67] | 100.00  [77.19;100.00] |
|  | Sensitivity % | 100.00  [87.54;100.00] | 100.00  [87.54;100.00] | 100.00  [87.54;100.00] | 100.00  [87.54;100.00] | 100.00  [87.54;100.00] |
| **BaseTyper™**  **BasePurifier™** | **Paired** |  |  |  |  |  |
|  | Specificity % | 100.00  [77.19;100.00] | 100.00  [77.19;100.00] | 100.00  [77.19;100.00] | 100.00  [77.19;100.00] | 100.00  [77.19;100.00] |
|  | Sensitivity % | 100.00  [87.54;100.00] | 100.00  [87.54;100.00] | 100.00  [87.54;100.00] | 100.00  [87.54;100.00] | 100.00  [87.54;100.00] |
|  | **Universal** |  |  |  |  |  |
|  | Specificity % | 92.3  [66.69;98.63] | 92.3  [66.69;98.63] | 100.00  [77.19;100.00] | 100.00  [77.19;100.00] | 100.00  [77.19;100.00] |
|  | Sensitivity % | 100.00  [87.54;100.00] | 100.00  [87.54;100.00] | 100.00  [87.54;100.00] | 100.00  [87.54;100.00] | 100.00  [87.54;100.00] |
