# Supplementary material for: One-instrument, objective microsatellite instability analysis using high-resolution melt
Source: PLoS One. 2024 Apr 25;19(4):e0302274. doi: 10.1371/journal.pone.0302274 (PMC11045061; doi:10.1371/journal.pone.0302274)
Supplement: S6 Table — (DOCX) [file pone.0302274.s006.docx]

**S6 Table. Sensitivity and specificity of paired samples and universal reference for five laboratories analyzing the same samples.**

|  |  | **BAT25** | **BAT26** | **NR22** | **NR24** | **MONO27** |
| --- | --- | --- | --- | --- | --- | --- |
| **Laboratory 1, 3, 4** | **Paired** |  |  |  |  |  |
|  | Specificity % | 100.00  [86.68;100.00] | 100.00  [86.68;100.00] | 100.00  [86.68;100.00] | 100.00  [86.68;100.00] | 100.00  [86.68;100.00] |
|  | Sensitivity % | 100.00  [56.55;100.00] | 100.00  [56.55;100.00] | 100.00  [56.55;100.00] | 100.00  [56.55;100.00] | 100.00  [56.55;100.00] |
|  | **Universal** |  |  |  |  |  |
|  | Specificity % | 96.00  [80.46;99.29] | 100.00  [86.68;100.00] | 100.00  [86.68;100.00] | 100.00  [86.68;100.00] | 100.00  [86.68;100.00] |
|  | Sensitivity % | 100.00  [56.55;100.00] | 100.00  [56.55;100.00] | 100.00  [56.55;100.00] | 100.00  [56.55;100.00] | 100.00  [56.55;100.00] |
| **Laboratory 2** | **Paired** |  |  |  |  |  |
|  | Specificity % | 100.00  [86.68;100.00] | 100.00  [86.68;100.00]) | 100.00  [86.68;100.00] | 100.00  [86.68;100.00] | 100.00  [86.68;100.00] |
|  | Sensitivity % | 100.00  [56.55;100.00] | 100.00  [56.55;100.00] | 100.00  [56.55;100.00] | 100.00  [56.55;100.00] | 100.00  [56.55;100.00] |
|  | **Universal** |  |  |  |  |  |
|  | Specificity % | 92.00  [75.03;97.78] | 100.00  [86.68;100.00] | 100.00  [86.68;100.00] | 96.00  [80.46;99.29] | 100.00  [86.68;100.00] |
|  | Sensitivity % | 100.00  [56.55;100.00] | 100.00  [56.55;100.00] | 100.00  [56.55;100.00] | 100.00  [56.55;100.00] | 100.00  [56.55;100.00] |
| **Laboratory 5** | **Paired** |  |  |  |  |  |
|  | Specificity % | 100.00  [86.68;100.00] | 100.00  [86.68;100.00] | 100.00  [86.68;100.00] | 100.00  [86.68;100.00] | 100.00  [86.68;100.00] |
|  | Sensitivity % | 100.00  [56.55;100.00] | 100.00  [56.55;100.00] | 100.00  [56.55;100.00] | 100.00  [56.55;100.00] | 100.00  [56.55;100.00] |
|  | **Universal** |  |  |  |  |  |
|  | Specificity % | 96.00  [80.46;99.29] | 100.00  [86.68;100.00] | 100.00  [86.68;100.00] | 100.00  [86.68;100.00] | 96.00  [80.46;99.29] |
|  | Sensitivity % | 100.00  [56.55;100.00] | 100.00  [56.55;100.00] | 100.00  [56.55;100.00] | 100.00  [56.55;100.00] | 100.00  [56.55;100.00] |
