# Supplementary material for: One-instrument, objective microsatellite instability analysis using high-resolution melt
Source: PLoS One. 2024 Apr 25;19(4):e0302274. doi: 10.1371/journal.pone.0302274 (PMC11045061; doi:10.1371/journal.pone.0302274)
Supplement: S9 Table — (DOCX) [file pone.0302274.s009.docx]

**S9 Table. 3x3 table from Laboratory 4 using paired samples.**

|  | | **MicroSight® MSI** | | |
| --- | --- | --- | --- | --- |
|  |  | **MSS** | **MSI-L** | **MSI-H** |
| **Lab. 4** | **MSS** | 13 | 1 | 0 |
|  | **MSI-L** | 4 | 1 | 0 |
|  | **MSI-H** | 0 | 0 | 11 |
