# Supplementary material for: One-instrument, objective microsatellite instability analysis using high-resolution melt
Source: PLoS One. 2024 Apr 25;19(4):e0302274. doi: 10.1371/journal.pone.0302274 (PMC11045061; doi:10.1371/journal.pone.0302274)
Supplement: S12 Table — (DOCX) [file pone.0302274.s012.docx]

**S12 Table. Deviating cases of paired samples from the validation cohort.**

|  |  | **Fragment analysis** | | | | | | | | | | | **MicroSight® MSI** | | | | | |
| --- | --- | --- | --- | --- | --- | --- | --- | --- | --- | --- | --- | --- | --- | --- | --- | --- | --- | --- |
| **Laboratory 2** | Sample# | BAT25 | | BAT26 | | DS123 | | D5S346 | | D17S50 | | Category | BAT25 | BAT26 | NR22 | NR24 | MONO27 | Category |
|  | 2 | S | | S | | I | | S | | S | | MSI-L | S | S | S | S | S | MSS |
|  | 25 | S | | S | | S | | S | | S | | MSS | S | I | I | I | S | MSI-H |
|  | 27 | S | | S | | I | | S | | S | | MSI-L | S | S | S | S | S | MSS |
| **Laboratory 3** | Sample# | BAT25 | | BAT26 | | NR21 | | NR24 | | NR27 | | Category | BAT25 | BAT26 | NR22 | NR24 | MONO27 | Category |
|  | 28 | S | | S | | I | | S | | S | | MSI-L | S | S | S | S | S | MSS |
| **Laboratory 4** | Sample# | BAT25 | | BAT26 | | NR21 | | NR24 | | MONO27 | | Category | BAT25 | BAT26 | NR22 | NR24 | MONO27 | Category |
|  | 2 | I | | S | | S | | S | | S | | MSI-L | S | S | S | S | S | MSS |
|  | 4 | I | | S | | S | | S | | S | | MSI-L | S | S | S | S | S | MSS |
|  | 5 | I | | S | | S | | S | | S | | MSI-L | S | S | S | S | S | MSS |
|  | 26 | S | | S | | S | | S | | S | | MSS | S | S | I | S | S | MSI-L |
| **Laboratory 5** | Sample# | BAT25 | BAT26 | D2S123 | D17S250 | D5S346 | BAT40 | D18S58 | NR21 | NR24 | TGFβII | Category | BAT25 | BAT26 | NR22 | NR24 | MONO27 | Category |
|  | 19 | S | S | I | S | S | S | S | S | S | S | MSI-L | S | S | S | S | S | MSS |
|  | 20 | S | S | I | S | S | S | S | S | S | S | MSI-L | S | S | S | S | S | MSS |
|  | 22 | S | S | S | I | S | S | S | S | S | S | MSI-L | S | S | S | S | S | MSS |
